# A Targeted Constitutive Mutation in the Apc Tumor Suppressor Gene Underlies Mammary But Not Intestinal Tumorigenesis

**DOI:** 10.1371/journal.pgen.1000547

**Published:** 2009-07-03

**Authors:** Claudia Gaspar, Patrick Franken, Lia Molenaar, Cor Breukel, Martin van der Valk, Ron Smits, Riccardo Fodde

**Affiliations:** 1 Department of Pathology, Josephine Nefkens Institute, Erasmus MC, Rotterdam, The Netherlands; 2 Human and Clinical Genetics Center, LUMC, Leiden, The Netherlands; 3 Department of Pathology, Netherlands Cancer Institute, Amsterdam, The Netherlands; Stanford University School of Medicine, Howard Hughes Medical Institute, United States of America

## Abstract

Germline mutations in the adenomatous polyposis coli (*APC*) gene are responsible for familial adenomatous polyposis (FAP), an autosomal dominant hereditary predisposition to the development of multiple colorectal adenomas and of a broad spectrum of extra-intestinal tumors. Moreover, somatic *APC* mutations play a rate-limiting and initiating role in the majority of sporadic colorectal cancers. Notwithstanding its multifunctional nature, the main tumor suppressing activity of the *APC* gene resides in its ability to regulate Wnt/β-catenin signaling. Notably, genotype–phenotype correlations have been established at the *APC* gene between the length and stability of the truncated proteins encoded by different mutant alleles, the corresponding levels of Wnt/β-catenin signaling activity they encode for, and the incidence and distribution of intestinal and extra-intestinal tumors. Here, we report a novel mouse model, *Apc*1572T, obtained by targeting a truncated mutation at codon 1572 in the endogenous *Apc* gene. This hypomorphic mutant allele results in intermediate levels of Wnt/β-catenin signaling activation when compared with other *Apc* mutations associated with multifocal intestinal tumors. Notwithstanding the constitutive nature of the mutation, *Apc* ^+/1572T^ mice have no predisposition to intestinal cancer but develop multifocal mammary adenocarcinomas and subsequent pulmonary metastases in both genders. The histology of the *Apc*1572T primary mammary tumours is highly heterogeneous with luminal, myoepithelial, and squamous lineages and is reminiscent of metaplastic carcinoma of the breast in humans. The striking phenotype of *Apc* ^+/1572T^ mice suggests that specific dosages of Wnt/β-catenin signaling activity differentially affect tissue homeostasis and initiate tumorigenesis in an organ-specific fashion.

## Author Summary

Although signal transduction pathways are often described as “on–off” systems, the more quantitative aspects of signalling are likely to represent a very important means of regulation of the downstream biological outcomes. Mutations in members of the canonical Wnt signaling pathway represent among the most common defects in human cancers. However, it is yet unclear which factors determine tissue and organ specificity of the tumours arising upon Wnt constitutive activation. Previously, we have generated a series of hypomorphic alleles at the *Apc* tumor suppressor gene and showed that the differentiation potential of embryonic stem cells is dependent on the dosage of Wnt/β-catenin signalling these mutants encode for. Likewise, analysis of the two mutational hits occurring at the Apc gene in human and mouse intestinal tumors showed that these are selected to retain specific residual dosages of β-catenin downregulation. Here, we provide further support for this “just-right” signalling model by targeting a germline *Apc* mutation encoding for very low levels of Wnt signalling activation when compared with other mutants known to trigger intestinal tumorigenesis. Notwithstanding the constitutive nature of this mutation, heterozygous mice show a remarkable and highly penetrant predisposition to multifocal and metastatic mammary cancers without the GI tract tumor phenotype characteristic of the majority of *Apc* mouse models.

## Introduction

Epithelial malignancies such as colorectal and breast cancer are thought to arise and progress towards malignancy due to alterations in signal transduction pathways that regulate the balance between self-renewal and differentiation in adult stem cell compartments [1]. The canonical Wnt/β-catenin signal transduction pathway plays a rate-limiting role in embryonic and adult stem cell renewal, and its aberrant activation is among the most common signaling defect in human cancers [2]. Activation of the canonical Wnt pathway leads to intracellular β-catenin stabilization and its translocation to the nucleus where it interacts with members of the Tcf/Lef family of transcription factors to modulate the expression of specific Wnt target genes (http://www.stanford.edu/~rnusse/pathways/targets.html). In the gastro-intestinal tract, Wnt/β-catenin signaling regulates stemness and differentiation of epithelial cells along the crypt-villus axis [3],[4]. Accordingly, truncating mutations in the *APC* tumor suppressor gene, the main negative regulator of the Wnt/β-catenin pathway, result in the constitutive activation of canonical Wnt signaling thus affecting stem cell differentiation and trigger tumor formation in the GI-tract and in other extra-intestinal tissues in a dosage-dependent fashion in man and mouse [5]–[8].

**Figure 1:**
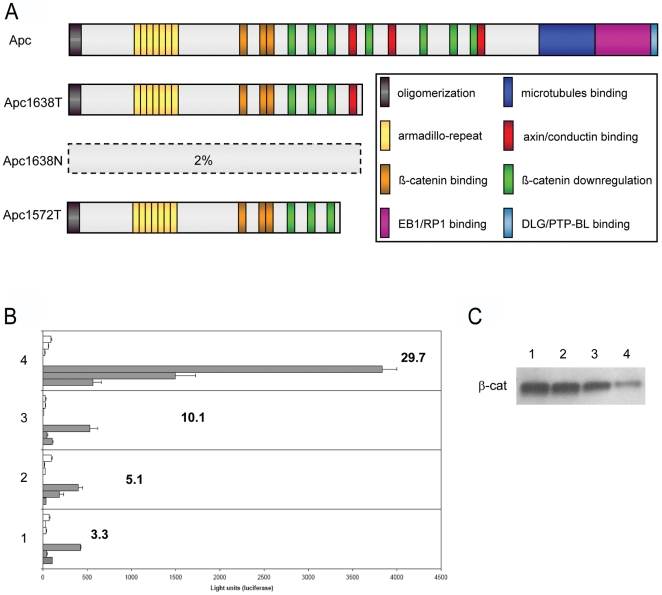
Biochemical characterization of the targeted *Apc*1572T allele. (A) Schematic representation of the APC tumor suppressor protein, its functional domains, and the truncated proteins resulting from the *Apc*1572T, *Apc*1638N, and *Apc*1638T targeted alleles. Only residual amounts (2%) of the truncated Apc1638N protein are encoded by the targeted allele, as shown by immuno-precipitation analysis of *Apc* ^1638N/1638N^ ES lines [5]. (B) β-catenin/TCF reporter assay (TOP-FLASH) analysis of *Apc* ^+/+^ (1) and *Apc*-mutant ES cell lines: Apc^1638T/1638T^ (2); Apc^1572T/1572T^ (3); Apc^1638N/1638N^ (4). Each bar represents the average measurement of the luciferase units from triplicate assays. For each cell line, 3 independent experiments were performed with the TOP (filled bars) and FOP (empty bars) reporter constructs. The bold figures represent the average TOP/FOP ratio of all independent experiments. Depicted error bars correspond to standard deviation. In brief, ES cells were plated on dishes coated with MEFs and subsequently transfected by lipofection with either the TOP-FLASH or FOP-FLASH reporter constructs [10] together with the Renilla luciferase vector for normalization purposes. (C) Immuno-precipitation (IP) analysis of Apc-bound β-catenin in *Ap*c-mutant ES cell lines. For comparative purposes, immuno-precipitates obtained from equal amounts of total cellular lysates were loaded.

The structure and distribution of β-catenin binding and downregulating motifs along the *APC* tumor suppressor gene is particularly suited to study the effects of specific dosages of canonical Wnt signaling on the multiplicity and tissue-specific distribution of the resulting tumors (Figure 1A). The vast majority of *APC* mutations found in hereditary and sporadic colorectal cancers are distributed in the 5′ half of the gene and are predicted to encode for stable truncated proteins encompassing up to 3 β-catenin downregulating (20 a.a.) domains. Stable truncation of the mouse *Apc* gene at codon 1638 as encoded by the *Apc* ^1638T^ allele, results in a protein retaining a sufficient number of functional domains to ensure wild type β-catenin regulation, namely 3 of the 7 β-catenin down-regulating domains and one Axin-binding SAMP repeat (Figure 1A) [9]. *Apc* ^+/1638T^ animals are tumor-free and even homozygous *Apc* ^1638T/1638T^ mice are viable with no apparent predisposition to tumorigenesis [9], in sharp contrast with the marked tumor predisposition and embryonic lethality characteristic of all *Apc*-mutant mouse models described to date in hetero- and homozygosity, respectively [6]. Notably, *Apc* ^1572T^, a targeted allele designed to truncate Apc immediately upstream of the only SAMP (Ser-Ala-Met-Pro) repeat encompassed by *Apc* ^1638T^ (Figure 1A), is characterized by an intermediate level of Wnt/β-catenin signaling activation, higher than wild type *Apc* and *Apc* ^1638T^ though significantly lower than other *Apc* targeted alleles known to result in GI tract tumors [9]. Here, we show that *Apc* ^+/1572T^ mice are characterized by a striking predisposition to multifocal mammary adenocarcinomas with no susceptibility to intestinal adenomas.

## Results

### The *Apc*1572T allele results in intermediate Wnt/β-catenin signaling levels and differentiation defects in embryonic stem cells

To allow the biochemical and functional characterization of the Wnt/β-catenin signaling defect encoded by the *Apc*1572T allele [9], we established *Apc* ^1572T/1572T^ embryonic stem (ES) cells from pre-implantation blastocysts and compared them by TopFLASH reporter assays [10] with *Apc* ^+/+^, *Apc* ^1638T/1638T^, and *Apc* ^1638N/1638N^ ES lines [5] (Figure 1B). The results show that *Apc* ^1572T/1572T^ ES cells encode for intermediate Wnt/β-catenin signaling levels, in between those characteristic of *Apc* ^1638N/1638N^ and *Apc* ^1638T/1638T^. The latter are in fact very close to those of wild type (*Apc* ^+/+^) ES cells, as previously reported [9]. These differences in Wnt/β-catenin signaling dosage are likely to result from diminished efficiency of β-catenin downregulation by the Apc1572T truncated protein due to the deletion of the only Axin-binding SAMP domain encompassed by Apc1638T. Immuno-precipitation (IP) analysis of the Apc-bound β-catenin fractions in the different *Apc*-mutant ES cell lines confirmed that, when compared with wild type (*Apc* ^+/+^) cells, decreasing amounts of Apc-bound β-catenin are observed in *Apc* ^1638T/1638T^, *Apc* ^1572T/1572T^, and *Apc* ^1638N/1638N^ ES cells (Figure 1C).

**Figure 2:**
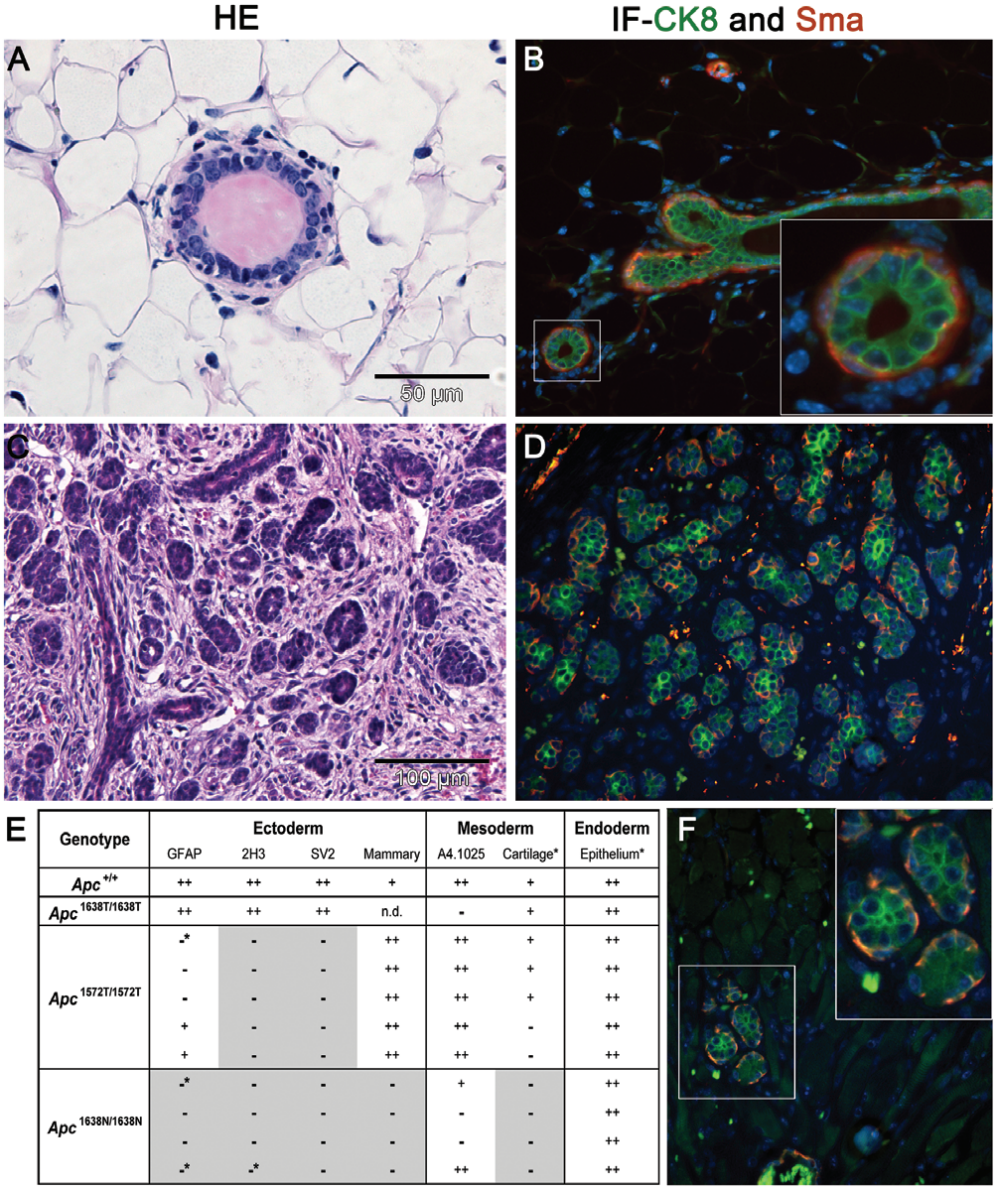
Teratoma formation assays indicate an intermediate differentiation defect in *Apc* ^1572T/1572T^ ES cells. (A) HE analysis of normal mammary gland with luminal cells surrounded by a basal layer of myoepithelial cells. (B) IF analysis of normal mammary glands for Ck8 (luminal cells, green) and Sma (myoepithelial, red). (C) HE staining of *Apc* ^1572T/1572T^ teratoma showing the typical mammary gland architecture with lobular and ductal structures. (D) IF analysis of *Apc* ^1572T/1572T^ teratomas for luminal and myoepithelial cell types. The frequency of these structures in teratomas derived from *Apc* ^1572T/1572T^ ES cells is largely increased when compared with (F) teratomas derived from *Apc* ^+/+^ ES cells. (E) Summary of the results of the teratoma differentiation assays of *Apc*-mutant ES cells. Antibodies employed to evaluate differentiation are: Glial Fibrillary Acidic Protein (GFAP) for glial cells; 2H3 for neurofilaments; SV2 for synaptic vesicles; A4.1025 for adult myosin. Mammary gland structures were primarily identified by HE and then confirmed by IF as shown in panels a–d; also in the case of cartilage and epithelial structures HE stained section were employed. n.d. not determined. Differentiation levels were scored as: (−) not present; (−*) vestigial presence; (+) present; (++) highly abundant. The shaded areas indicate groups of teratomas for which the corresponding antibody staining was negative.

Previously, we showed that different levels of β-catenin signaling affect the ability of mouse embryonic stem (ES) cells to differentiate towards specific lineages in a dosage-dependent fashion [5]. To address the same question for the *Apc*1572T allele, we have subcutaneously injected undifferentiated *Apc* ^1572T/1572T^ ES cells into syngenic mice to induce formation of teratomas, as previously described [5]. The differentiation profiles of the *Apc* ^1572T/1572T^ teratomas were then investigated by histological and immuno-histochemical analysis, and compared with those obtained with wild type (*Apc* ^+/+^) and other *Apc*-mutant ES cells. In line with their intermediate level of constitutive Wnt/β-catenin signaling activation, *Apc* ^1572T/1572T^ teratomas show a more heterogeneous spectrum of ecto-, meso-, and endodermal lineages than *Apc* ^1638N/1638N^ (characterized by a higher TopFLASH reporter activity; see Figure 1B), though still more limited in their differentiation capacity than *Apc* ^1638T/1638T^ (characterized by TopFLASH reporter activity comparable with wild type ES cells) (Figure 2E). In agreement with previous observations [5], several differentiation types, namely neural, bone, cartilage and ciliated epithelia were absent in *Apc* ^1638N/1638N^ teratomas. In particular, markers employed to identify neuroectodermal lineages did not stain *Apc* ^1638N/1638N^ sections, in contrast with *Apc* ^1572T/1572T^ teratomas where a limited but significant number of the cells were GFAP positive. Differentiation to striated muscle was also severely affected and detectable in only a minority of the *Apc* ^1638N/1638N^ sections [5], whereas all *Apc* ^1572T/1572T^ teratomas analyzed revealed positive myosin staining. Notably, among the cell types positively identified in *Apc* ^+/+^ teratomas, mammary epithelia were relatively more abundant in *Apc* ^1572T^ sections, as shown by the combined staining with SMA and CK8 and the typical tissue architecture with luminal cells on top of a myoepithelial basal layer (Figure 2A–2D and Figure 2F). Hence, homozygous *Apc* ^1572T^ ES cells are characterized by an intermediate differentiation defect between *Apc* ^1638N/1638N^ and *Apc* ^1638T/1638T^, with an unusual enrichment in mammary epithelial differentiation.

### Homozygosity for *Apc* ^1572T^ results in embryonic lethality

*Apc* ^+/1572T^ mice were generated from two independent ES clones previously obtained by targeting a hygromycin cassette at codon 1572 of the endogenous mouse *Apc* gene [9]. To assess the post-natal viability of *Apc* ^1572T/1572T^ mice, heterozygous *Apc* ^+/1572T^ animals were interbred and four litters analyzed (n = 18 mice). None of the resulting animals was found to be homozygous for the targeted allele (p = 0.0034, χ^2^ test) indicating that the *Apc* ^1572T^ allele results in embryonic lethality, as previously observed for the majority of *Apc*-mutant mouse models with the only exception of *Apc* ^1638T^ [6],[9]. Thus, the difference between the Apc^1572T^ and Apc^1638T^ truncated proteins, namely the Axin-binding SAMP motif pinpoints to a key role for this functional domain in Wnt signaling regulation during embryonic development.

### *Apc* ^+/1572T^ mice develop mammary adenocarcinomas encompassing heterogeneous cell lineages

Phenotypic analysis of *Apc* ^+/1572T^ heterozygous animals was performed on a total of 69 mice and compared with wild type, *Apc* ^+/1638N^ and *Apc* ^+/Min^ on two different genetic backgrounds, namely inbred C57Bl6/J and F1 C57Bl6/J×129Ola (Table 1). GI tumor multiplicities and localization in *Apc* ^+/Min^ and *Apc* ^+/1638N^ did not differ from previously published data [11],[12]. Notably, *Apc* ^+/1572T^ mice do not have an increased susceptibility to intestinal tumors when compared with wild type animals. Epidermal cysts and desmoids, previously shown in the *Apc*1638N model [12], were also observed among *Apc* ^+/1572T^ mice with the same gender-specific distribution though with diminished multiplicity and penetrance (Table 1).

**Table 1:** Overview of the phenotypic comparisons between *Apc* ^+/1572T^ and other *Apc*-mutant mouse models.

B	*Genotype*	Sex	Age^1^ (months) Mean (Range)	Incidence of pyloric tumors	Incidence GI tumours^2^	Multiplicity GI tumours^3^ Mean±SD (Range)	Incidence desmoids	Multiplicity desmoids Mean±SD (Range)	Incidence cysts	Multiplicity cysts Mean±SD (Range)	Incidence mammary AdCa	Incidence Liver tumors
F1	*Apc* ^+/+^ (n = 28)	F (9)	7.7 (4.1–15.1)	0	0	n.d.	0	n.d.	0	n.d.	0	0
		M (19)	12.8 (4.7–18.6)	0	0	n.d.	0	n.d.	0	n.d.	0	0
	*Apc* ^+/Min^ (n = 12)	F (6)	6.2 (4.6–7.5)	1/6 (16.6%)	6/6 (100%)	34.4±7.4 (29–47)	2/5 (40%)	1±1.4 (0–3)	5/5 (100%)	1.8±0.4 (1–2)	1/6 (16.6%)	0
		M (5)	6.3 (4.7–10.4)	3/5 60%	5/5 100%	37.4±28.5 (8–82)	3/4 (75%)	1.75±1.5 (0–3)	4/4 (100%)	2.25±0.5 (2–3)	0	0
	*Apc* ^+/1638N^ (n = 27)	F (14)	14.6 (13–16.4)	14/14 (100%)	13/14 (92.9%)	4.3±3.3 (0–11)	13/13 100%	14.3±6.6 (3–25)	13/13 (100%)	9.7±4.1 (4–17)	0	0
		M (13)	14.3 (12.3–16.3)	13/13 (100%)	12/13 (92.3%)	5.2±3.1 (0–11)	13/13 (100%)	72.9±23.0 (48–119)	13/13 (100%)	50.2±14.2 (25–71)	0	0
	*Apc* ^+/1572T^ (n = 20)	F (7)	8.1 (3.3–11.7)	0	0	n.d.	3/6 (50%)	1.8±2.6 (0–6)	3/6 (50%)	2.5±3.5 (0–7)	6/7 (85.7%)	0
		M (13)	16.9 (11.6–21)	7/13 (53.8%)	1*/13 (7.7%)	n.d. (0–1)	11/11 (100%)	45.5±31.8 (6–110)	7/11 (63.6%)	8.8±9.8 (0–27)	4/13 (30.8%)	4 (30.8%)
Ola	*Apc* ^+/+^ (n = 10)	F (5)	11 (8.9–15.6)	0/5	0/5	n.d.	n.d.	n.d.	n.d.	n.d.	0	0
		M (5)	18.3 (16.7–19.4)	0/5	3/5 (60%)	n.d. (0–1)	n.d.	n.d.	n.d.	n.d.	0	0
	*Apc* ^+/1572T^ (n = 32)	F (18)	8.2 (4.5–10.9)	0/18	0/18	n.d.	n.d.	n.d.	n.d.	n.d.	17/18 (94.4%)	2/18 (11.1%)
		M (14)	12.4 (4.6–17.9)	1/14 (7%)	4/14 (28.5%)	n.d. (0–1)	n.d.	n.d.	n.d.	n.d.	7/14 (50%)	4/14 (28.6%)
B6	*Apc* ^+/1572T^ (n = 17)	F (10)	4.8 (2.6–15.1)	0/10	0/10	n.d.	n.d.	n.d.	n.d.	n.d.	10/10 (100%)	0/10
		M (7)	15.2 (14.2–16.1)	0//7	0/7	n.d.	n.d.	n.d.	n.d.	n.d.	2/7 (28.6%)	2/7 (28.6%)

Notes: Incidence is given as percentage of affected animals.

1,3 Animals were sacrificed when signs of discomfort were apparent and/or when tumor size reached 2 cm.

2 The incidence of GI tumors was calculated after exclusion of the pyloric lesions as these present in clusters often difficult to count.

3 The multiplicity of GI tumors was calculated based on all animals with the exception of those where the high tumor burden made the count not feasible.

*: This specific animal was found to carry a single tumor at 21 months of age, likely to represent a sporadic case. Background (B) of the different strains analyzed: F1: C57Bl6/J x 129Ola; Ola: inbred 129Ola; B6: inbred C57Bl6/J. n.d. not determined.

**Figure 3:**
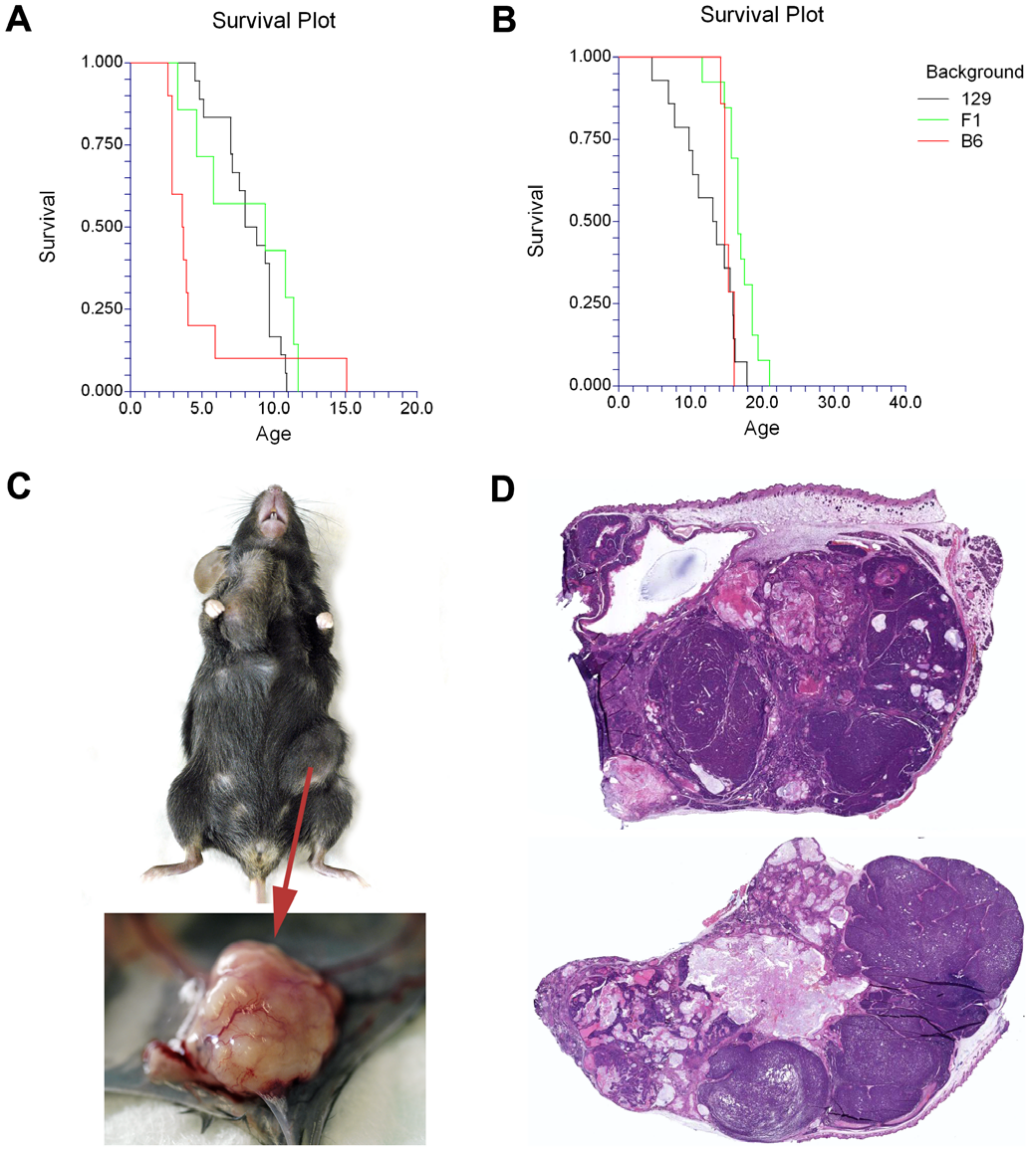
Phenotypic characterization of *Apc* ^+/1572T^ mice: mammary adenocarcinomas are composed by mixed differentiation lineages with heterogeneous patterns of β-catenin intracellular accumulation and subcellular localization. Survival curves of (A) female and (B) male *Apc* ^+/1572T^ mice, respectively. The black, green and red lines are representative of mice in the 129Ola, F1 B6x129Ola, and B6 respectively. Please note that in these graphs, age of death represents the moment at which, due to the presence of signs of discomfort or because the tumor size exceeded 2 cm^3^, mice had to be euthanized according to institutional and national regulations. (C) Macroscopic image of the appearance of the mammary adenocarcinomas characteristic of the *Apc*1572T model. (D) Examples of global digital microscopy scans of two mammary adenocarcinomas from *Apc* ^+/1572T^ mice illustrative of the multi-lineage nature of these lesions.

**Figure 4:**
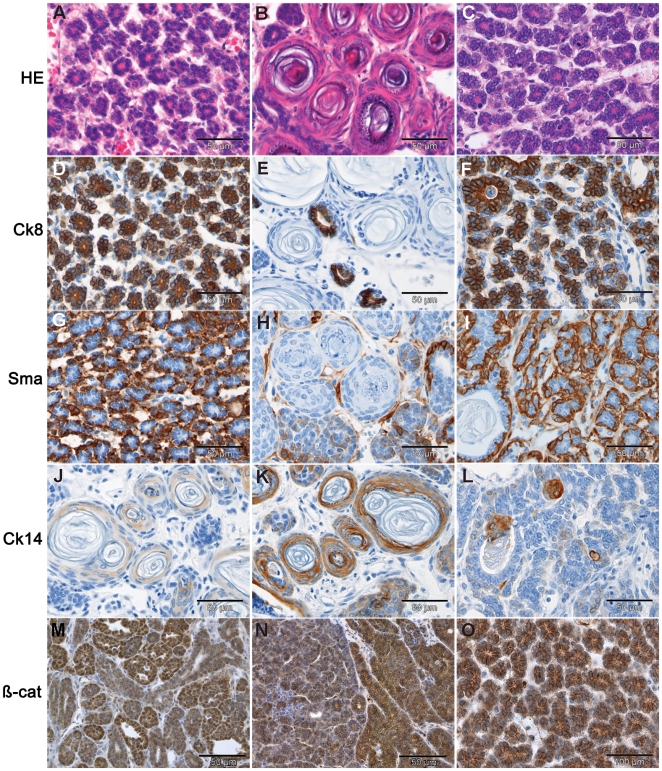
Differentiation. HE staining of mammary tumors (A–B) and pulmonary metastases (C) from *Apc* ^+/1572T^ mice shows typical mammary glandular architecture and squamous differentiation. (D–F) Luminal epithelial differentiation as shown by cytokeratin 8 (Ck8) IHC staining. (G–I) Myoepithelial differentiation revealed by IHC staining with the Sma antibody. (J–L) IHC analysis with antibodies directed against cytokeratin 14 (Ck14) confirm the presence of squamous differentiation (hair follicle and skin cellular types). (M–O) β-catenin IHC analysis shows heterogeneous subcellular localization and intracellular accumulation with fewer cells characterized by positive nuclear staining. The results shown in this figure were confirmed in 12 independent primary tumors.

Together with the absence of intestinal tumors, the most striking phenotypic feature of the *Apc* ^+/1572T^ mouse model is undoubtedly represented by the highly penetrant incidence of multifocal mammary tumors among virgin females (100%) and males (30%), in sharp contrast with *Apc* ^+/Min^ and *Apc* ^+/1638N^ animals (1/6 and 0/14, respectively) (Table 1, Figure 3A–3D). These tumors typically arise around 3 months of age in C57BL6/J animals, though age of onset fluctuates in the different genetic backgrounds (Table 1). Histological analysis of the *Apc* ^+/1572T^ mammary tumors revealed a lobular arrangement with both acinar and glandular growth patterns (Figure 4A). Varying degrees of squamous metaplasia were observed in all tumors analyzed. These structures resemble skin and hair follicle differentiation (Figure 4B), in some cases strikingly similar to that observed in trichoepithelioma originated from the hair follicle. This highly heterogeneous histology with diffuse lobular hyperplasia and different degrees of squamous metaplasia was also present in smaller lesions. Thus, trans-differentiation of mammary epithelial cells takes place at an early stage during *Apc*–driven tumorigenesis. Immunohistochemistry (IHC) analysis revealed that all *Apc* ^+/1572T^ mammary adenocarcinomas (n = 12) encompass luminal and myoepithelial cell types together with areas of squamous metaplasia (Figure 4D–4E, Figure 4G–4H, and Figure 4J–4K). Heterogeneous patterns of β-catenin subcellular localization were also observed upon IHC analysis of *Apc* ^+/1572T^ mammary tumors with the majority of parenchymal cells showing membrane-bound and cytoplasmatic staining along with smaller patches characterized by strong nuclear staining (Figure 4M–4N).

**Figure 5:**
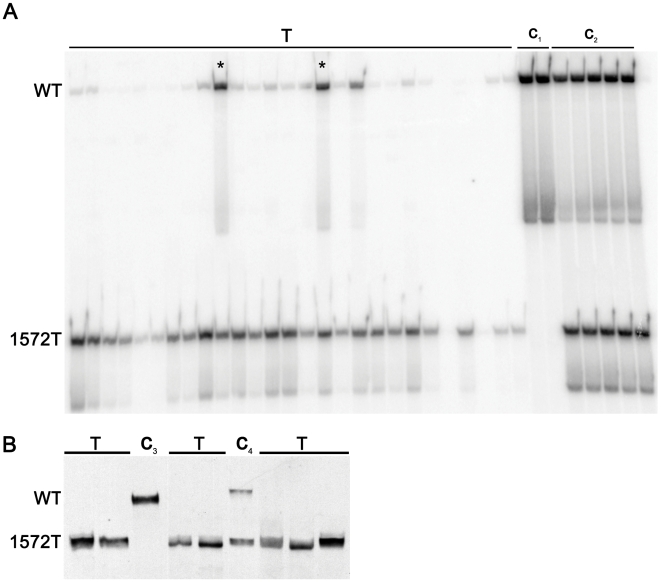
LOH analysis of *Apc* ^+/1572T^ mammary adenocarcinomas. (A) PCR–based LOH analysis of tumour DNA samples amplified in the presence of radioactive nucleotides as previously described [35]. Samples were scored as having lost the wild type allele when the ratio between the intensity of the two alleles was ≤0.6. *Apc* ^+/1572T^ mammary tumor samples (T); *Apc* ^+/+^ DNA control (C_1_); *Apc* ^+/1572T^ DNA control (C_2_). Out of the 27 samples (collected from 23 tumours) analysed, only two (lanes marked by an asterisks) show a ratio higher than 0.6 and were accordingly scored as not having allelic imbalance. (B) Western analysis of tumour-derived total protein lysates confirms the somatic loss of full length Apc. Tumour samples (T); wild type control (C_3_); *Apc* ^+/1572T^ control from tail sample (C_4_).

As observed in the vast majority of the intestinal and extra-intestinal tumors caused by *APC* gene mutations in man and mouse, LOH analysis of DNA and protein samples from *Apc* ^+/1572T^ mammary tumors revealed allelic imbalance in more than 90% of cases (21/23) (Figure 5A). These observations were validated by western analysis of tumor-derived cell lysates (Figure 5B).

During necropsy, *Apc* ^+/1572T^ mice were identified with gross pulmonary alterations subsequently identified as metastases of the primary mammary adenocarcinomas by histological and IHC analysis. Similar to the primary mammary carcinomas, these lesions encompassed both luminal and myoepithelial cell types (Figure 4C, 4F, and 4I). Areas of squamous differentiation were also present, though significantly less abundant than in the primary mammary tumors (Figure 4L). β-catenin IHC analysis of the *Apc* ^+/1572T^ lung metastases recapitulated the staining pattern of the primary tumors (Figure 4O).

### *Apc* ^+/1572T^/*Smad4* ^+/Sad^ mice regain predisposition to intestinal tumors

**Figure 6:**
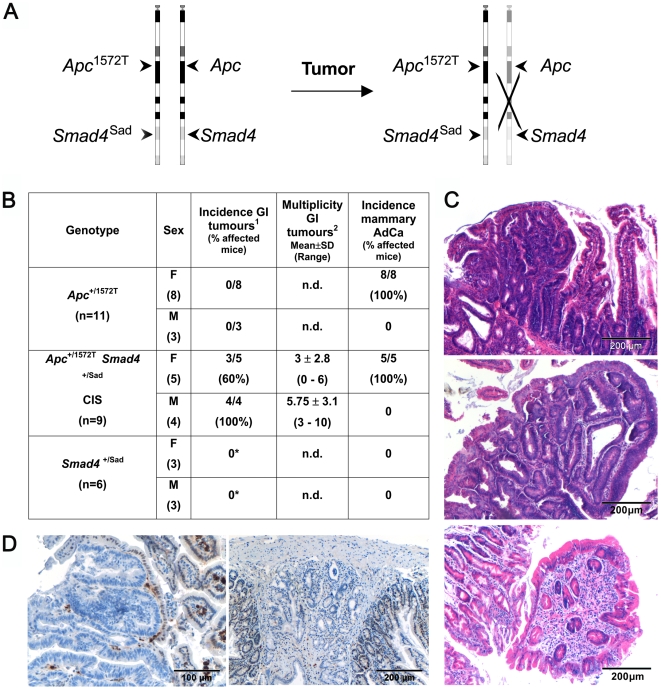
Phenotypic and molecular analysis of the compound *in cis Apc* ^+/1572T^/*Smad4* ^+/Sad^ mouse model. (A) Schematic illustration of the chr. 18 LOH event in intestinal tumors from *in cis Apc* ^+/1572T^/*Smad4* ^+/Sad^ mice leading to loss of both *Smad4* and *Apc* wild-type alleles. (B) Comparative phenotypic analysis of the intestinal and mammary tumor predisposition among *Apc* ^+/1572T^, *Smad4* ^+/Sad^, and *Apc* ^+/1572T^/*Smad4* ^+/Sad^ mice. *Notes*: (1) The incidence of GI tumors was calculated after exclusion of the pyloric lesions as these present in clusters often difficult to count. (2) The multiplicity of GI tumors was calculated based on all animals with the exception of those where the high tumor burden made the count not feasible. The asterisks indicate that the apparent absence of intestinal tumor in *Smad4* ^+/Sad^ control animals is not in contradiction with what previously published. These mice were sacrificed at time points matched with the ages at which compound *Apc* ^+/1572T^/*Smad4* ^+/Sad^ mice had to be sacrificed due to the high GI and mammary tumor burden (♀: 90.4 days +/−28.4; ♂: 118.5 days +/−26.2). However, in *Smad4* ^+/Sad^ animals the majority of the tumors appear at 9 months of age [15]. (C) H&E staining of intestinal tumor sections from *Apc* ^+/1572T^ (top), *Apc* ^+/1572T^/*Smad4* ^+/Sad^ (middle), and *Smad4* ^+/Sad^ (bottom) mice. (D) Smad4 IHC analysis of two intestinal adenomas from *Apc* ^+/1572T^/*Smad4* ^+/Sad^ mice showing loss of Smad4 expression. LOH was observed in 100% of the polyps (n = 15) analyzed. PCR–based LOH analysis of the same cohort of *Apc* ^+/1572T^/*Smad4* ^+/Sad^ polyps revealed loss of wild-type *Apc* allele in 87% of the cases (13/15; data not shown).

To provide additional experimental support for the “just right” signaling model for *Apc*-driven mammary tumorigenesis, we have taken advantage of a recent study according to which Tgf-β signaling antagonizes canonical Wnt signaling thus negatively regulating stem cell self-renewal [13]. Hence, Tgf-β alterations such as those resulting from *Smad4* loss of function mutations, are expected to lead to a further increase of Wnt/β-catenin signaling in the *Apc*-mutant cellular background. Therefore, we have bred *Apc* ^+/1572T^ animals with *Smad4* ^+/Sad^, a mouse model for juvenile polyposis previously developed in our laboratory [14]. *Smad4* ^+/Sad^ animals are characterized by a late-onset predisposition to hyperplastic intestinal polyps which develop in the absence of a 2^nd^ hit at either the *Smad4* or the *Apc* locus [15]. As both these tumor suppressor genes map to chromosome 18 in the mouse, we have generated *Apc* ^+/1572T^/*Smad4* ^+/Sad^ compound heterozygous mice where both targeted alleles are in the *in cis* phase on chr. 18 as previously described for the *Apc*1638N model [15]. As shown in Figure 6A, *Apc* ^+/1572T^/*Smad4* ^+/Sad^ mice show a similar incidence of mammary adenocarcinomas similarly to *Apc* ^+/1572T^, but are characterized by multiple GI-tract tumors. These polyps are of the adenomatous type and become apparent at a much earlier age than the hyperplastic lesions with a pronounced stromal component characteristic of the *Smad4* ^+/Sad^ model (Figure 6B). Moreover, the vast majority of the *Apc* ^+/1572T^/*Smad4* ^+/Sad^ intestinal polyps show loss of the entire chr. 18 carrying the wild type alleles of both tumor suppressor genes [15] (Figure 6C), whereas the intestinal lesions characteristic of the *Smad4* ^+/Sad^ mice retain the wild type *Smad4* allele at first, and show *Smad4* LOH (but not at the *Apc* locus) only at more advanced progression stages [15]. Although it cannot be excluded that loss of *Smad4* function underlies intestinal tumour formation in these animals through Tgf-β/BMP downstream effectors independent of Wnt signaling, the histology and molecular features of the *Apc* ^+/1572T^/*Smad4* ^+/Sad^ GI polyps strongly suggest that the further increase of Wnt/β-catenin signaling conferred by the *Smad4* mutation in the *Apc*-mutant background results in intestinal tumours in the compound mice without apparently affecting the mammary cancer phenotype.

## Discussion

Although the role of Wnt/β-catenin signalling has been established for a broad spectrum of cancers [2], it is yet unclear which factors determine tissue and organ specificity of the tumors arising upon its constitutive activation. In familial adenomatous polyposis (FAP) for example, different *APC* germline mutations lead to different spectra of extra-colonic manifestations depending on their localization along the gene and on the stability of the resulting truncated polypeptide [16],[17]. In general, it appears that more hypomorhic *APC* mutants localized at the 5′ and 3′ ends of the gene result in atypical FAP phenotypes characterized by reduced intestinal adenoma multiplicities and enhanced tumorigenesis outside the GI tract (mainly desmoids and cutaneous cysts) [18]. Here, we show that a hypomorphic mutation in the mouse *Apc* tumour suppressor gene results in a highly penetrant predisposition to mammary adenocarcinomas without the intestinal tumours characteristic of FAP patients carrying germline *APC* mutations and of most *Apc*-mutant mouse models reported to date [6]. This unique tumor phenotype is even more accentuated by the presence of pulmonary metastases arising from the primary mammary lesions, a feature rarely observed in genetically modified mouse models of epithelial malignancies.

Notably, DU4475, a human breast cancer cell line derived from a recurrent thoracic wall tumor following mastectomy due to a poorly differentiated invasive ductal carcinoma [19], carries a nonsense mutation at codon 1577 of the *APC* gene, only 5 residues downstream of the targeted *Apc*1572T allele [20],[21]. Hence, it is plausible to think that only very specific alterations result in the “just right” level of Wnt/β-catenin signaling activation and trigger neoplastic transformation in the mammary gland, presumably by affecting self-renewal of the stem cell population as shown by the observed metaplastic changes. In this hypothetical model, the critical Wnt signalling threshold level to ensure stem cell homeostasis, i.e. the equilibrium between self-renewal and differentiation, is considerably lower in the mammary gland than in the intestinal epithelium. However, this would imply that most *Apc-*mutant mouse models, characterized by a pronounced predisposition to multiple intestinal tumors [6], should also be susceptible to mammary tumorigenesis. Indeed, most targeted *Apc* models show predisposition to mammary adenocarcinomas, though with considerably less penetrance than the GI tract tumors [22]–[24]. More importantly, transplantation of mammary glands from *Apc* ^+/Min^ mice into wild type recipient animals results in metaplastic adenocarcinomas [22], thus showing that the propensity to develop mammary tumors is intrinsic to the *Apc*-mutant cells.

Further confirmation of the validity of the “just right” signaling model has been more recently provided by the conditional inactivation of both *Apc* alleles in the lactating mammary gland cells (by *BLG*-*Cre*) which resulted in multiple metaplastic growths which do not progress to neoplasia [25]. Even more support for the “just right” signaling hypothesis has been delivered by the study by Kuraguchi et al. where the conditional loss of a single *Apc*-LoxP allele is specifically driven in mammary progenitor cells (by *K14-Cre*) and in lactating luminal cells (by *WAP-Cre*) [26]. Only the *K14-Cre*-mediated *Apc* heterozygosity resulted into mammary adenocarcinomas with similar histological features to those observed in *Apc* ^+/1572T^ tumors, thus suggesting the early progenitor or stem cell of origin of these mixed lineage tumors. Notably, analysis of the wild type *Apc* allele in the *K14-Cre*;*Apc* ^+/CKO^ mammary tumors revealed the presence of specific somatic point mutations clustering in the codon 1521–1570 region, i.e. very close to residue 1572 where our own mutation was targeted [26]. Thus, the genetic mechanisms underlying *Apc*-mediated mammary tumor formation are strikingly similar between the tissue specific conditional knock-out *K14-Cre*;*Apc* ^+/CKO^ model and the constitutive *Apc* ^+/1572T^ mice: in both cases one allele is completely lost (the germline conditional KO allele in *K14-Cre*;*Apc* ^+/CKO^ and the somatic loss in *Apc* ^+/1572T^) whereas the other retains residual β-catenin downregulating activity (the somatic point mutations found in *K14-Cre*;*Apc* ^+/CKO^, and the targeted germline mutation in *Apc* ^+/1572T^). The final outcome of these selection processes is the retention of the dosage of Wnt/β-catenin signaling that is “just right’ to allow clonal expansion of mammary stem cells or early progenitors and their neoplastic transformation.

A second implication of the “just right” signalling model is that an increase of the Wnt/β-catenin signaling dosage conferred by the *Apc*1572T mutation is expected to trigger intestinal tumor formation. Recently, it has been reported that Tgf-β signaling antagonizes canonical Wnt thus negatively regulating stem cell self-renewal [13]. Hence, Tgf-β alterations such as those resulting from *Smad4* loss of function mutations, are expected to lead to a further increase of Wnt/β-catenin signaling in the *Apc*-mutant cellular background. Accordingly, *Apc* ^+/1572T^/*Smad4* ^+/Sad^ compound heterozygous mice revealed a similar incidence of mammary adenocarcinomas as in *Apc* ^+/1572T^, but were also characterized by multiple GI-tract tumors never observed in the parental strain, even when kept for longer than 1 year. These polyps arise from loss of the entire mouse chr. 18 where both the *Apc* and *Smad4* tumor suppressor genes map, and are clearly different from the hyperplastic lesions characteristic of *Smad4* ^+/Sad^ mice which retain the wild type *Smad4* allele [15]. Although it cannot be excluded that loss of *Smad4* function underlies intestinal tumour formation in these animals through Tgf-β/BMP downstream effectors independent of Wnt signaling, the histology and molecular features of the *Apc* ^+/1572T^/*Smad4* ^+/Sad^ GI polyps strongly suggest that the further increase of Wnt/β-catenin signaling conferred by the *Smad4* mutation in the *Apc*-mutant background results in the observed predisposition to intestinal tumours in the compound mice without apparently affecting the mammary cancer phenotype.

In view of the well known multifunctional nature of the APC tumor suppressor protein [27], one could also envisage that functional motifs other that those binding and downregulating β-catenin and Axin, could underlie the striking tumor phenotype of the *Apc*1572T model. However, with the only exception of the SAMP motif, all the known functional domains located in the COOH third of the protein which are truncated by the targeted mutation at codon 1572 are also absent in the *Apc*1638T model, previously shown to be tumor free even when bred to homozygosity [9].

Several members of the Wnt signaling cascade including the Wnt1 ligand and β-catenin, have been shown to result in mammary hyperplasia and tumors when overexpressed in transgenic mice in a tissue-specific fashion [26], [28]–[31]. Notably, the MMTV-driven Wnt1 and β-catenin transgenic models develop mammary metaplasia highly reminiscent of the tumors observed in *Apc* ^+/1572T^ animals, especially as far as the heterogeneity of their histology is concerned. Among the broad spectrum of breast cancers observed in man, the histological subtype that most closely resembles the *Apc*1572T mammary adenocarcinomas is represented by metaplastic carcinoma of the squamous type, responsible for 1 to 5% of the total breast cancer burden [32]. Notably, genetic alterations in different members of the Wnt signaling pathway including *CTNNB1* (β-catenin), *APC*, and *WISP3* (Wnt1 Induced Secreted Protein 3) are relatively common among metaplastic carcinomas of the breast [33]. Hence, it is plausible to think that only very specific gene alterations result in the “just right” level of Wnt/β-catenin signaling activation to trigger neoplastic transformation in the human mammary gland. On the contrary to other, more common gene mutations leading to breast cancer (e.g. ErbB2) which are likely to affect more committed progenitor cells, Wnt/β-catenin signaling activation presumably affects self-renewal and differentiation capacity of the mammary primordial stem cell or in very early progenitors as shown by the metaplastic changes and the presence of both myoepithelial and luminal lineages in Wnt-driven mammary tumors.

Apart from their resemblance with breast metaplastic carcinomas in man, the mammary adenocarcinomas observed in *Apc* ^+/1572T^ mice without the concomitant presence of multiple intestinal polyps raise the possibility that a fraction of the hereditary breast cancer cases could be caused by germline *APC* mutations located in the proximity of codon 1572. We are currently testing this by sequencing the *APC* gene around codons 1500–1700 in hereditary breast cancer patients which tested negative for *BRCA1*/*BRCA2* germline mutations. Also, we are searching whether FAP patients with *APC* germline mutations in the proximity of codon 1600 show any eventual predisposition to breast cancer. However, it is also possible that mutations in other members of the Wnt pathway may result in the “just right” signaling level and in tumor predispositions similar to the *Apc*1572T model.

In conclusion, we have shown that a targeted *Apc* mutation encoding for intermediate levels of Wnt/β-catenin signaling results in a highly penetrant predisposition to multifocal mammary adenocarcinomas without the intestinal tumors characteristic of most *Apc*-mutant mouse models and individuals carrying germline *APC* mutations. Our results, also supported by several mammary cancer studies in mouse and man suggests that only specific dosages of canonical Wnt signaling are “just right” to expand the mammary stem/progenitor cell and result into mixed lineage (metaplastic) tumorigenesis.

## Materials and Methods

### Mouse strains

*Apc* ^+/1572T^ mice were generated from two independent 129Ola ES clones targeted at codon 1572 as previously described [9]. Stable expression of the truncated protein encoded by the targeted allele was confirmed by western blot analysis (not shown). Chimeras were bred with 129Ola and C57Bl6/J animals to generate inbred 129Ola, and F1 (C57Bl6/J×129Ola) *Apc* ^+/1572T^ mice. To generate inbred C57Bl6/J *Apc* ^+/1572T^ mice, F1 (C57Bl6/J×129Ola) animals were backcrossed to inbred C57Bl6/J mice for at least 8 generations. Control *Apc* ^1638N^ and *Apc* ^Min^ animals were generated by crossing inbred C57Bl6/J mutants with 129Ola for comparative purposes. Heterozygous mice were employed for the phenotypic characterization, together with wild type littermates as controls. Compound *Apc* ^+/1572T^/*Smad4* ^+/Sad^ *in cis* mice were generated as previously described [15].

All animals were fed *ad-libitum* and housed in SPF facilities. Animal experiments were performed according to institutional and national regulations.

### ES cell lines generation and teratoma differentiation assays

*Apc* ^1572T/1572T^ ES cells were derived from pre-implantation (3.5 dpc) blastocysts as previously described [34]. Teratomas were obtained upon subcutaneous injection of 5×10^6^ cells into isogenic mice.

### β-catenin/TCF reporter assays

5×10^5^ ES cells were plated on dishes coated with MEFs (mouse embryonic fibroblasts) and subsequently transfected by Lipofectamine 2000 (Invitrogen) with either 500ng of the TOP-FLASH or FOP-FLASH reporter constructs [10] together with 5ng of the Renilla luciferase vector for normalization purposes. Luciferase activity was measured by Dual–Luciferase Reporter Assay System (Promega).

### Immunoprecipitation (IP) and western analysis

Apc IP analysis was performed according to previously published protocol [9] using the AFPN polyclonal antibody. Detection of Apc and β-catenin in the destruction complex was carried out by using the following antibodies: Apc Ab1 (OP44, Oncogene), β-catenin (610154, BD Biosciences).

### Immunohistochemistry (IHC) and immunofluorescence

Tissues were fixed in PFA (4%) and embedded in paraffin. Five μm sections were mounted on slides stained by HE for routine histology. Antibodies employed for IHC analysis include: β-catenin (1∶2000, 1247-1, Epitomics), Troma1 which recognizes a Ck8 epitope (1∶400, Hybridoma Bank), Sma (1∶200, M0851, DakoCytmomation), Ck14 (1∶10000, PRB-155P, Covance), Ck6 (1∶5000, PRB-169P, Covance). The Ck6 and Ck14 antibodies are employed to detect hair follicle and skin differentiation, respectively. However, when employed at a lower dilution (1∶1000), Ck6 also detects mammary epithelial cells. All IHC images presented here were obtained with higher dilution (1∶5000) aiming at the identification of squamous differentiation lineages. The same primary antibody dilutions were employed for IF analysis, rabbit anti-rat-FITC (Sigma) and goat anti-mouse-A594 (Invitrogen) were used for signal detection.

### LOH analysis

LOH analysis for the Apc locus was performed as previously described [35]. In brief, tumour sections were obtained from *Apc* ^+/1572T^ mammary adenonarcinomas and stained by HE. Tumour areas were localized and microdissected by LCM (Laser Capture Microdissection; Leica Microssystems), followed by DNA isolation. PCR-amplified fragments were resolved in a denaturing 6% polyacrylamide gel, dried on paper and the number of counts per allele was determined on a phosphor imager. Subsequently, the number of counts of the larger allele was divided by the counts of the smaller allele to obtain an allelic ratio. A mean allelic ratio was calculated for at least five normal controls. This value was used to generate a comparative ratio (CR) >1.0 by dividing the tumor allelic ratio by the mean normal allelic ratio. In this way, normalization of imbalances already observed in wild type controls due to preferential amplification of a specific allele, is not necessary. A CR ≥1.5 was interpreted as significant, i.e. indicative of loss of the wild type allele.

These PCR-based observations were further confirmed by western blot analysis. Mammary tumors and control tail tissue samples were digested with Blendzyme3 (Roche Diagnostics) in DMEM medium supplemented with gentamycin. Protein lysates, separation and blotting were performed using the NuPage Gel System (gel Tris-Acetate 3–8%) according to manufacturer's protocol (Invitrogen). Apc detection was accomplished using the antibody Apc Ab1 (OP44, Oncogene).

LOH analysis for the *Smad4* gene was carried out exclusively at the protein level by IHC on tissue sections (1∶100, sc-7966, Santa Cruz). In this case, antigen retrieval was performed with Tris-EDTA pH 8.0 and the signal was detected using Envision HRP-ChemMate Kit (DAKO).

## References

[1] Reya T, Morrison SJ, Clarke MF, Weissman IL (2001). Stem cells, cancer, and cancer stem cells.. Nature.

[2] Reya T, Clevers H (2005). Wnt signalling in stem cells and cancer.. Nature.

[3] Batlle E, Henderson JT, Beghtel H, van den Born MM, Sancho E (2002). Beta-catenin and TCF mediate cell positioning in the intestinal epithelium by controlling the expression of EphB/ephrinB.. Cell.

[4] van de Wetering M, Sancho E, Verweij C, de Lau W, Oving I (2002). The beta-catenin/TCF-4 complex imposes a crypt progenitor phenotype on colorectal cancer cells.. Cell.

[5] Kielman MF, Rindapaa M, Gaspar C, van Poppel N, Breukel C (2002). Apc modulates embryonic stem-cell differentiation by controlling the dosage of beta-catenin signaling.. Nat Genet.

[6] Fodde R, Smits R, Clevers H (2001). APC, signal transduction and genetic instability in colorectal cancer.. Nat Rev Cancer.

[7] Gaspar C, Fodde R (2004). APC dosage effects in tumorigenesis and stem cell differentiation.. Int J Dev Biol.

[8] Albuquerque C, Breukel C, van der Luijt R, Fidalgo P, Lage P (2002). The ‘just-right’ signaling model: APC somatic mutations are selected based on a specific level of activation of the beta-catenin signaling cascade.. Hum Mol Genet.

[9] Smits R, Kielman MF, Breukel C, Zurcher C, Neufeld K (1999). Apc1638T: a mouse model delineating critical domains of the adenomatous polyposis coli protein involved in tumorigenesis and development.. Genes Dev.

[10] Korinek V, Barker N, Morin PJ, van Wichen D, de Weger R (1997). Constitutive transcriptional activation by a beta-catenin-Tcf complex in APC−/− colon carcinoma.. Science.

[11] Su LK, Kinzler KW, Vogelstein B, Preisinger AC, Moser AR (1992). Multiple intestinal neoplasia caused by a mutation in the murine homolog of the APC gene.. Science.

[12] Smits R, van der Houven van Oordt W, Luz A, Zurcher C, Jagmohan-Changur S (1998). Apc1638N: a mouse model for familial adenomatous polyposis-associated desmoid tumors and cutaneous cysts.. Gastroenterology.

[13] Falk S, Wurdak H, Ittner LM, Ille F, Sumara G (2008). Brain area-specific effect of TGF-beta signaling on Wnt-dependent neural stem cell expansion.. Cell Stem Cell.

[14] Hohenstein P, Molenaar L, Elsinga J, Morreau H, van der Klift H (2003). Serrated adenomas and mixed polyposis caused by a splice acceptor deletion in the mouse Smad4 gene.. Genes Chromosomes Cancer.

[15] Alberici P, Jagmohan-Changur S, De Pater E, Van Der Valk M, Smits R (2006). Smad4 haploinsufficiency in mouse models for intestinal cancer.. Oncogene.

[16] Eccles DM, van der Luijt R, Breukel C, Bullman H, Bunyan D (1996). Hereditary desmoid disease due to a frameshift mutation at codon 1924 of the APC gene.. Am J Hum Genet.

[17] van der Luijt RB, Meera Khan P, Vasen HF, Breukel C, Tops CM (1996). Germline mutations in the 3′ part of APC exon 15 do not result in truncated proteins and are associated with attenuated adenomatous polyposis coli.. Hum Genet.

[18] Fodde R, Khan PM (1995). Genotype-phenotype correlations at the adenomatous polyposis coli (APC) gene.. Crit Rev Oncog.

[19] Langlois AJ, Holder WD, Iglehart JD, Nelson-Rees WA, Wells SA (1979). Morphological and biochemical properties of a new human breast cancer cell line.. Cancer Res.

[20] Schlosshauer PW, Brown SA, Eisinger K, Yan Q, Guglielminetti ER (2000). APC truncation and increased beta-catenin levels in a human breast cancer cell line.. Carcinogenesis.

[21] van de Wetering M, Barker N, Harkes IC, van der Heyden M, Dijk NJ (2001). Mutant E-cadherin breast cancer cells do not display constitutive Wnt signaling.. Cancer Res.

[22] Moser AR, Mattes EM, Dove WF, Lindstrom MJ, Haag JD (1993). ApcMin, a mutation in the murine Apc gene, predisposes to mammary carcinomas and focal alveolar hyperplasias.. Proc Natl Acad Sci U S A.

[23] Moser AR, Hegge LF, Cardiff RD (2001). Genetic background affects susceptibility to mammary hyperplasias and carcinomas in Apc(min)/+ mice.. Cancer Res.

[24] van der Houven van Oordt CW, Smits R, Schouten TG, Houwing-Duistermaat JJ, Williamson SL (1999). The genetic background modifies the spontaneous and X-ray-induced tumor spectrum in the Apc1638N mouse model.. Genes Chromosomes Cancer.

[25] Gallagher RC, Hay T, Meniel V, Naughton C, Anderson TJ (2002). Inactivation of Apc perturbs mammary development, but only directly results in acanthoma in the context of Tcf-1 deficiency.. Oncogene.

[26] Kuraguchi M, Ohene-Baah NY, Sonkin D, Bronson RT, Kucherlapati R (2009). Genetic mechanisms in apc-mediated mammary tumorigenesis.. PLoS Genet.

[27] Fodde R (2003). The multiple functions of tumour suppressors: it's all in APC.. Nat Cell Biol.

[28] Nusse R, van Ooyen A, Cox D, Fung YK, Varmus H (1984). Mode of proviral activation of a putative mammary oncogene (int-1) on mouse chromosome 15.. Nature.

[29] Tsukamoto AS, Grosschedl R, Guzman RC, Parslow T, Varmus HE (1988). Expression of the int-1 gene in transgenic mice is associated with mammary gland hyperplasia and adenocarcinomas in male and female mice.. Cell.

[30] Michaelson JS, Leder P (2001). beta-catenin is a downstream effector of Wnt-mediated tumorigenesis in the mammary gland.. Oncogene.

[31] Imbert A, Eelkema R, Jordan S, Feiner H, Cowin P (2001). Delta N89 beta-catenin induces precocious development, differentiation, and neoplasia in mammary gland.. J Cell Biol.

[32] Rosen PP, Ernsberger D (1987). Low-grade adenosquamous carcinoma. A variant of metaplastic mammary carcinoma.. Am J Surg Pathol.

[33] Hayes MJ, Thomas D, Emmons A, Giordano TJ, Kleer CG (2008). Genetic changes of wnt pathway genes are common events in metaplastic carcinomas of the breast.. Clin Cancer Res.

[34] Joyner AL, Sedivy JM (2000). Gene targeting: a practical approach. Oxford.

[35] Smits R, Kartheuser A, Jagmohan-Changur S, Leblanc V, Breukel C (1997). Loss of Apc and the entire chromosome 18 but absence of mutations at the Ras and Tp53 genes in intestinal tumors from Apc1638N, a mouse model for Apc-driven carcinogenesis.. Carcinogenesis.

